# Can a multicomponent multidisciplinary implementation package change physicians’ and nurses’ perceptions and practices regarding thrombolysis for acute ischemic stroke? An exploratory analysis of a cluster-randomized trial

**DOI:** 10.1186/s13012-019-0940-0

**Published:** 2019-11-27

**Authors:** Md Golam Hasnain, Christopher R. Levi, Annika Ryan, Isobel J. Hubbard, Alix Hall, Christopher Oldmeadow, Alice Grady, Amanda Jayakody, John R. Attia, Christine L. Paul

**Affiliations:** 1 School of Medicine and Public Health (SMPH), University of Newcastle (UoN), Callaghan, NSW Australia; 2 The Sydney Partnership for Health, Education, Research & Enterprise (SPHERE), Liverpool, NSW Australia; 3 Hunter Medical Research Institute (HMRI), New Lambton Heights, NSW Australia; 4 Hunter New England Local Health District, Population Health, Wallsend, NSW Australia; 5 Priority Research Centre for Health Behaviour, University of Newcastle, Callaghan, NSW Australia; 6 John Hunter Hospital, New Lambton Heights, NSW Australia

## Abstract

**Background:**

The Thrombolysis ImPlementation in Stroke (TIPS) trial tested the effect of a multicomponent, multidisciplinary, collaborative intervention designed to increase the rates of intravenous thrombolysis via a cluster randomized controlled trial at 20 Australian hospitals (ten intervention, ten control). This sub-study investigated changes in self-reported perceptions and practices of physicians and nurses working in acute stroke care at the participating hospitals.

**Methods:**

A survey with 74 statements was administered during the pre- and post-intervention periods to staff at 19 of the 20 hospitals. An exploratory factor analysis identified the structure of the survey items and linear mixed modeling was applied to the final survey domain scores to explore the differences between groups over time.

**Result:**

The response rate was 45% for both the pre- (503 out of 1127 eligible staff from 19 hospitals) and post-intervention (414 out of 919 eligible staff from 18 hospitals) period. Four survey domains were identified: (1) hospital performance indicators, feedback, and training; (2) personal perceptions about thrombolysis evidence and implementation; (3) personal stroke skills and hospital stroke care policies; and (4) emergency and ambulance procedures. There was a significant pre- to post-intervention mean increase (0.21 95% CI 0.09; 0.34; *p* < 0.01) in scores relating to hospital performance indicators, feedback, and training; for the intervention hospitals compared to control hospitals. There was a corresponding increase in mean scores regarding perceptions about the thrombolysis evidence and implementation (0.21, 95% CI 0.06; 0.36; *p* < 0.05). Sub-group analysis indicated that the improvements were restricted to nurses’ responses.

**Conclusion:**

TIPS resulted in changes in some aspects of nurses’ perceptions relating to the evidence for intravenous thrombolysis and its implementation and hospital performance indicators, feedback, and training. However, there is a need to explore further strategies for influencing the views of physicians given limited statistical power in the physician sample.

**Trial registration:**

ACTRN12613000939796, UTN: U1111–1145-6762.

Contributions to the literature

- This study demonstrated the ability of a multicomponent implementation intervention to influence the perceptions of clinical staff regarding hospital performance indicators, feedback, and training; and their individual perceptions about the evidence base supporting poststroke thrombolysis and its implementation.
- This change occurred in the context of a transient change in the proportion of patients receiving thrombolysis for acute stroke.
- This study illustrates the importance of exploring processes as well as outcomes in the context of implementation trials, in that a change in clinician perceptions was achieved alongside a transient change in clinical practice. This suggests the need to further explore the perceptions/practice nexus during implementation trials.

## Background

Implementation of evidence-based recommendations is critical to delivering optimal clinical care to patients and achieving improvements in health outcomes [1]. However, the adoption of such recommendations into clinical practice often faces barriers [2] at the individual, organizational, and system levels [3].

Acute ischemic stroke (AIS) is one of the leading causes of mortality and morbidity globally [3] and in Australia [4]. Intravenous thrombolysis can improve clinical outcomes if administered within 4.5 h of symptom onset to eligible patients with AIS [5]. However, despite evidence of its efficacy and despite its inclusion in clinical guidelines [6], thrombolysis rates have remained persistently low over the last 10 years, at around 11% among all stroke cases [4]. Several potential barriers restricting the administration of intravenous thrombolysis in patients experiencing AIS [7] have been identified. At the individual level, Shiffman et al. demonstrated that physicians’ knowledge of the desired behavior could lead to improvements across quality indicators in emergency care [8]. Similarly, improved skills and opportunities to perform the desired behavior led to increased staff confidence in the care of patients with chronic cardiac failure [9]. Additionally, individual staff attitudes were associated with adherence to clinical guidelines [10]. In the case of AIS, the strong evidence base has been shown to increase physicians’ level of certainty about the impact and administration of intravenous thrombolysis [11, 12], and their familiarity with, and motivation to adhere to, recommended guidelines [7, 13]. At the health systems level, resources and a supportive workplace environment have been shown to positively influence evidence-based practice [3]. Inefficient in-hospital processes for managing emergency stroke patients and a lack of appropriate infrastructure, staffing, and hospital capacity are known barriers to poor rates of intravenous thrombolysis [7, 13]. As both physicians and nurses have a central role in thrombolysis, their perceptions and practices potentially influence intravenous thrombolysis rates [14, 15].

The Thrombolysis ImPlementation in Stroke (TIPS) study investigated a multicomponent, multidisciplinary, collaborative intervention designed to increase rates of intravenous thrombolysis [16]. The TIPS intervention was developed using the Behaviour Change Wheel theoretical framework [16]. The study found significant differences in the rates of intravenous thrombolysis between intervention and control hospitals after 16 months of “active” intervention. However, after the end of the implementation package (assessed over a 12-month post-intervention period), the increased proportions of tPA in intervention hospitals were no longer significant [17]. Recognizing that the views of physicians and nurses are important to achieving practice change [18], the study included a pre- and post-intervention survey which aimed to assess attitudes toward thrombolysis and experiences of various barriers and enablers to thrombolysis implementation. Such data can provide important insights into the intermediate impacts of the intervention and help to shape future interventions aimed at increasing rates of intravenous thrombolysis. Physicians and nurses may face different tasks and challenges even in the same medical environment and therefore, their perceptions and attitudes toward patient care may vary [19]. Hence, it is critically important to explore perceptions and attitudes toward patient care from both professions. As there are known differences between metropolitan and non-metropolitan hospitals such as staff experience, workload, infrastructure, etc. [20], the location of hospitals should be considered when assessing staff perceptions. Staff perceptions and practices are often explored using study-specific, non-validated measures. Therefore, wherever possible, it is important to identify the validity of the measure; for example, factor analysis can be used to assess how well a new measure captures the intended construct [21].

Therefore, the study aimed to (i) assess the validity of the staff survey measure through the exploratory factor analysis of the survey and (ii) investigate whether the perceptions and practices of the physicians and nurses involved in the TIPS study changed as a result of the TIPS intervention and whether any changes were specific to the practice group (physician or nurse) and hospital location (metropolitan or non-metropolitan).

## Methods

### Design and setting

The TIPS study recruited 20 hospitals across three states of Australia between 2011 and 2015. It evaluated the effectiveness of a multicomponent, multidisciplinary, collaborative intervention aimed at improving rates of intravenous thrombolysis [16, 17]. TIPS study hospitals were randomized to either receive the intervention activities (intervention hospitals) or continue with usual care (control hospitals) [17]. This study includes two cross-sectional surveys: one during the pre-intervention (2012-mid 2013) and another during the post-intervention (2015) period. We used STrengthening the Reporting of OBservational studies in Epidemiology (STROBE) guideline to report the study (Additional file 1). The survey was anonymous and administered to physicians and nurses employed in the participating hospitals [16].

### TIPS intervention and its activities

Intervention components were developed in accordance with the behavior change wheel [16] and strategies with a preferred emphasis on the following components of behavior change wheel: education, persuasion, training, modeling, and enablement. Seven intervention components were delivered actively over 16 months which included pre-workshop meetings, collaborative communal workshops, site-based working groups, web-based training modules, regular telephone case monitoring, bi-monthly feedback of thrombolysis rate, and bi-monthly inter-site teleconferences. Intervention activities and the study timeline are described in Table 1.

**Table 1:** Framework of interventional activity with the timeline

Aug–Sep 2013	Sep 2013	Oct 2013	Jan–Dec 2014	March–Nov 2014	March–Nov 2014	March–Dec 2014	Meeting dates not on fixed period	Oct 2014
Executive buy-in (calls or visits)	Pre-workshop site meetings	Workshop 1	Online training modules	Bi-monthly teleconferences	Quarterly feedback on thrombolysis	Regular case monitoring	Site-based working group meetings	Workshop 2
	1. Situational analysis 2. Executive buy-in 3. Preliminary target setting	1. Goals and target setting 2. Review of current thrombolysis rate 3. Situational analysis 4. Barriers and solutions 5. Forming a site working group	1. Competency-based assessments 2. Clinical decision-making skills 3. Eligibility criteria for stroke thrombolysis	1. Intrahospital collaboration 2. Barriers and solutions	1. Motivation 2. Tracking against targets 3. Feedback	1. Motivation 2. Case review 3. Training update 4. Site update	1. Multidisciplinary collaboration within the hospital 2. Case reviews Target settings 3. Barriers and solutions	1. Review of current and new goals 2. Sustainability of change achieved 3. Planning for the future

### Participants

All participating hospitals had a Stroke Unit or the staffing equivalent, and all were in the early stages of implementing intravenous thrombolysis. Participating hospitals included those that were publicly and privately funded and metropolitan and non-metropolitan based. Metropolitan and non-metropolitan hospitals were defined according to the Australian Standard Geographical Classification Remoteness Areas [22]. Eligible survey respondents were physicians and nurses who worked in the Stroke Units and/or the Emergency departments of the participating hospitals and had a role in assessing or managing acute stroke patients during the survey time period.

### Procedure

Eligible survey participants were identified by a staff member within each hospital responsible for the organization of stroke care, and this was usually a nurse (survey coordinator). Eligible participants were invited to participate via a combination of email and personal communication. All surveys were completed in hard copy and these were deposited in collection boxes. The survey coordinator provided data on the estimated number of eligible staff, the number of surveys distributed, and the number returned.

### Outcome measures

The survey was made up of 74 statements and in the first section, respondents were asked to rate their agreement using a five-point Likert Scale: Strongly disagree, disagree, agree, strongly agree, and not applicable [23] and the second section had some participants and hospital related information. A copy of this survey has been added to this manuscript (Additional file 2: Supplement 1). Survey items were selected from the previously published literature on behavior change and implementation of evidence-based practice. In addition, the National Stroke Foundation’s Clinical Guidelines for the management of Stroke and its recommendation for hospital facilities and evidence for intravenous thrombolysis was also considered to finalize the survey items [24, 25]. The survey items were also piloted by a team of behavioral researchers, stroke clinicians, emergency physicians, and stroke nurses at the University of Newcastle and the Hunter New England Local Health District [26]. The survey was divided into two sections and they are as follows.

### Section A: stroke care and intravenous thrombolysis

This section was titled “Your Views on tPA” and included 60 statements in total. Of these, 11 were directed at physicians only, and seven were directed at nurses only. Statements investigated issues related to knowledge and skills in assessing AIS and eligible patients for intravenous thrombolysis, hospital policy, and performance indicators for stroke care, impact, safety, and barriers to intravenous thrombolysis, the hospital’s monitoring and feedback systems, its emergency service, the protocol used to identify, treat and follow-up patients with stroke, and staff skills levels and staff training facilities. An example of a Section A statement is: “This hospital has goals for improving performance in stroke care”.

### Section B: individual and hospital characteristics

This section was entitled “About You and Your Workplace” and included 14 questions. Of these, three were directed at physicians only, and one at nurses only. Questions investigated a respondent’s age, gender, role within the hospital, and the number of years worked in a stroke unit and/or stroke care. They also investigated a hospital’s intravenous thrombolysis rate, pre-arrival notification system from the ambulance, responsibilities around data entry, the proportion of stroke patients seen by Emergency physicians, number of patients referred to stroke care, proportion treated with intravenous thrombolysis, and the respondent’s role and responsibilities around intravenous thrombolysis. An example of a Section B statement is “Does the hospital have arrangements in place for pre-arrival notification of stroke patients from the ambulance service?”

### Statistical analysis

#### Identifying construct validity and internal consistency

The frequencies and percentages of the responses for each statement were examined. An exploratory factor analysis was conducted on all statements, except those with > 20% missing values or “not applicable” responses, as they were assessed as having limited relevance [27]. A principal factors method was undertaken to identify the underlying factor structure [28]. The number of factors to retain was determined by assessing the following criteria: (i) the Kaiser-Criteria (eigenvalue more than 1 rule); (ii) the break in the scree plot; and (iii) assessment of parallel analysis [29]. The number of factors suggested by these three criteria oblique rotation was done and the results of each assessed and compared to determine the most appropriate factor structure [29]. The final factor structure was determined as the structure that met the following criteria: items loading on only one factor ≥ 0.4, minimal cross-loadings between items [30], and the structure that makes conceptual sense (Additional file 2: Supplement 3). Finally, to check and measure the reliability and internal consistency of the final factor structure, the Cronbach’s alpha coefficient for each selected factor was calculated [31].

#### Calculation of scores

Following factor analysis, each selected factor was defined as a domain. Domain scores were calculated for each participant, by summing all statement responses in a domain and dividing them by the number of non-missing statements. The total score for each domain for each participant was four. Likert responses were allocated a score of strongly disagree = 1, disagree = 2, agree = 3, and strongly agree = 4. Only those who responded to at least 75% of the statements within each domain were calculated a domain score [32].

#### Measuring the effect of the intervention

Separate linear mixed models were conducted for each domain score to identify whether staff statements differed in response to the intervention. Between-group differences in the change in mean staff responses from pre-intervention to post-intervention were assessed. In each model, the main effect for the intervention group and time were included as fixed effects as well a group by time interaction term. Baseline thrombolysis rate was also included as a fixed effect in all models to control for this factor. A random intercept for the hospital was included to account for the clustered design of the trial. To assess our secondary aim, similar models were also conducted separately by profession (i.e., physician and nurse) and geographical location (i.e., metropolitan and non-metropolitan) to allow for assessment of these factors as potential effect modifiers. Due to violations in the assumption of homoscedasticity, robust errors were employed for models assessing domain 1 and 2 of staff barriers. For domain, three and four bootstrap estimation was employed due to violations in the assumption of normality.

## Results

All 20 TIPS hospitals were invited to participate in the staff survey; 19 hospitals participated in the pre-intervention survey and 18 of those participated in the post-intervention survey. During the pre-intervention period, of the 1127 eligible staff, 503 returned completed surveys, whereas during the post-intervention period, of the 919 eligible staff, 414 returned completed surveys, equating to a 45% response rate for both surveys. Table 2 reports respondents’ characteristics against the intervention and control hospitals and the pre- and post-intervention surveys.

**Table 2:** Difference in participants’ distribution and characteristics between intervention and control hospitals for both pre-intervention and post-intervention survey

	Pre-intervention 503 (55%)		Post-intervention 414 (45%)	
	Intervention 260 (52%)	Control 240 (48%)	Intervention 192 (49%)	Control 202 (51%)
Characteristics				
Age, *n* (%)				
≤ 25 years	24 (10)	17 (8)	17 (10)	13 (7)
> 25–45 years	149 (62)	117 (54)	101 (57)	109 (59)
> 45–60 years	60 (25)	76 (35)	50 (28)	58 (31)
> 60 years	9 (4)	5 (2)	8 (5)	6 (3)
Sex, *n* (%)				
Male	84 (34)	75 (32)	54 (29)	70 (36)
Female	164 (66)	160 (68)	130 (71)	125 (64)
Work experience in emergency/stroke, *n* (%)				
≤ 5 years	90 (35)	79 (34)	49 (27)	62 (32)
> 5–10 years	74 (29)	68 (30)	62 (34)	64 (33)
> 10–15 years	38 (15)	34 (15)	26 (14)	35 (18)
> 15 years	53 (21)	48 (21)	45 (25)	34 (17)
Staff type, *n* (%)				
Physician	74 (29)	69 (30)	47 (26)	67 (34)
Nurse	181 (71)	163 (70)	137 (74)	129 (66)
Distribution				
Location, *n* (%)				
Metropolitan	124 (48)	170 (71)	119 (62)	152 (75)
Regional	136 (52)	70 (29)	73 (38)	50 (25)
Baseline thrombolysis rate, *n* (%)				
Strata 1	106 (41)	134 (56)	82 (43)	123 (61)
Strata 2	127 (49)	76 (32)	83 (43)	63 (31)
Strata 3	27 (10)	30 (12)	27 (14)	16 (8)

Of the 48 statements included in the factor analysis, 33 statements were maintained representing four domains (Additional file 2: Supplement 2). Domain 1 included 14 statements related to individual and hospital-level performance indicators, feedback, and training. Domain 2 included nine statements related to individual-level perceptions about the evidence supporting thrombolysis and its implementation. Domain 3 included six statements related to staff stroke care skills and hospital stroke care policies. Domain 4 included four statements related to emergency and ambulance procedures. The Cronbach’s alphas for domain 1, 2, 3, and 4 were 0.90, 0.79, 0.80, and 0.85 respectively.

When comparing results between the control and intervention hospitals, the domain 1 mean score in the intervention hospitals showed a significant mean increase of 0.21 (95% CI 0.09; 0.34; *p* < 0.01) from the pre- to the post-intervention surveys (Table 3), indicating a positive change in staff perceptions relating to hospital performance indicators, feedback, and training. Similarly, the domain 2 mean score showed a significant increase of 0.21 (95% CI 0.06; 0.36; *p* < 0.05) (Table 4), indicating a positive change in staff perceptions relating to the evidence supporting thrombolysis and its implementation. The between-group differences for domains 3 and 4 were not significant indicating no change in staff perceptions relating to the individual’s level of stroke care skills, the hospital’s stroke care policies, and/or the emergency and ambulance procedures (Tables 5 and 6).

**Table 3:** Effect of intervention on domain 1 score = performance indicator, feedback and training

	Intervention group		Control group		Intervention vs. control group^a,b^; *p* value
	Pre-intervention (Mean ± SD)	Post-intervention (Mean ± SD)	Pre-intervention (Mean ± SD)	Post-intervention (Mean ± SD)	
Overall					
	2.95 ± 0.48	3.17 ± 0.47	3.03 ± 0.50	3.02 ± 0.46	0.21 (0.09; 0.34)**; 0.001
Location					
Metropolitan	3.07 ± 0.52	3.21 ± 0.47	3.04 ± 0.50	3.03 ± 0.49	0.15 (− 0.07; 0.37); 0.175
Non-metropolitan	2.83 ± 0.40	3.11 ± 0.47	2.99 ± 0.51	2.99 ± 0.41	0.26 (0.17; 0.35)***; 0.000
Job role					
Physician	2.94 ± 0.41	3.05 ± 0.45	2.99 ± 0.51	2.96 ± 0.41	0.12 (− 0.08; 0.31); 0.243
Nurse	2.95 ± 0.50	3.19 ± 0.47	3.07 ± 0.48	3.05 ± 0.46	0.25 (0.06; 0.44)*; 0.010

*p value < 0.05 considered as significant

**p value < 0.01 considered as significant

***p value < 0.001 considered as significant

^a^Change from pre-intervention to post-intervention survey

^b^Linear mixed model controlled for category based on baseline thrombolysis rate

**Table 4:** Effect of intervention on domain 2 score = perceptions about the evidence base for intravenous thrombolysis and its implementation

	Intervention group		Control group		Intervention vs. control group^a,b^; *p* value
	Pre-intervention (Mean ± SD)	Post-intervention (Mean ± SD)	Pre-intervention (Mean ± SD)	Post-intervention (Mean ± SD)	
Overall					
	3.18 ± 0.47	3.29 ± 0.41	3.24 ± 0.43	3.14 ± 0.49	0.21 (0.06; 0.36)*; 0.007
Location					
Metropolitan	3.20 ± 0.49	3.33 ± 0.38	3.25 ± 0.43	3.12 ± 0.51	0.25 (0.04; 0.46)*; 0.021
Non-metropolitan	3.16 ± 0.46	3.22 ± 0.44	3.23 ± 0.43	3.19 ± 0.46	0.09 (− 0.20; 0.38); 0.523
Job role					
Physician	3.18 ± 0.48	3.10 ± 0.50	3.17 ± 0.40	2.90 ± 0.57	0.19 (− 0.10; 0.48); 0.206
Nurse	3.19 ± 0.47	3.36 ± 0.34	3.28 ± 0.44	3.27 ± 0.39	0.18 (0.01; 0.36)*; 0.039

*p value < 0.05 considered as significant

**p value < 0.01 considered as significant

***p value < 0.001 considered as significant

^a^Change from pre-intervention to post-intervention survey

^b^Linear mixed model controlled for category based on baseline thrombolysis rate

**Table 5:** Effect of intervention on domain 3 score = personal stroke skills and hospital stroke care policies

	Intervention group		Control group		Intervention vs. control group^a,b^; *p* value
	Pre-intervention (Mean ± SD)	Post-intervention (Mean ± SD)	Pre-intervention (Mean ± SD)	Post-intervention (Mean ± SD)	
Overall					
	3.48 ± 0.45	3.60 ± 0.39	3.48 ± 0.47	3.55 ± 0.43	0.04 (− 0.10; 0.18); 0.597
Location					
Metropolitan	3.52 ± 0.45	3.65 ± 0.36	3.48 ± 0.47	3.59 ± 0.39	0.01 (− 0.10; 0.13); 0.828
Non-metropolitan	3.44 ± 0.45	3.52 ± 0.43	3.46 ± 0.47	3.46 ± 0.51	0.09 (−v0.14; 0.22); 0.601
Job role					
Physician	3.49 ± 0.43	3.63 ± 0.40	3.48 ± 0.48	3.57 ± 0.40	0.04 (− 0.14; 0.22); 0.696
Nurse	3.49 ± 0.45	3.59 ± 0.39	3.48 ± 0.47	3.54 ± 0.46	0.04 (− 0.13; 0.21); 0.670

*p value < 0.05 considered as significant

**p value < 0.01 considered as significant

***p value < 0.001 considered as significant

^a^Change from pre-intervention to post-intervention survey

^b^Linear mixed model controlled for category based on baseline thrombolysis rate

**Table 6:** Effect of intervention on domain 4 score = perceptions toward emergency service

	Intervention group		Control group		Intervention vs. control group^a,b^; *p* value
	Pre-intervention (Mean ± SD)	Post-intervention (Mean ± SD)	Pre-intervention (Mean ± SD)	Post-intervention (Mean ± SD)	
Overall					
	3.08 ± 0.61	3.40 ± 0.49	3.15 ± 0.58	3.36 ± 0.56	0.10 (− 0.07; 0.27); 0.178
Location					
Metropolitan	3.13 ± 0.58	3.44 ± 0.48	3.18 ± 0.54	3.34 ± 0.53	0.14 (− 0.09; 0.38); 0.219
Non-metropolitan	3.03 ± 0.63	3.34 ± 0.52	3.09 ± 0.66	3.41 ± 0.62	− 0.02 (− 0.36; 0.30); 0.860
Job role					
Physician	3.09 ± 0.67	3.13 ± 0.50	3.13 ± 0.55	3.26 ± 0.55	− 0.11 (− 0.38; 0.16); 0.398
Nurse	3.07 ± 0.58	3.50 ± 0.45	3.17 ± 0.57	3.41 ± 0.55	0.18 (0.02; 0.34)*; 0.041

*p value < 0.05 considered as significant

**p value < 0.01 considered as significant

***p value < 0.001 considered as significant

^a^Change from pre-intervention to post-intervention survey

^b^Linear mixed model controlled for category based on baseline thrombolysis rate

When comparing results between physicians and nurses, the domain 1, 2, and 4 mean scores in nurses showed a significant mean increase of 0.25 (95% CI 0.06; 0.44; *p* < 0.05), 0.18 (95% CI 0.01; 0.36; p < 0.05), and 0.18 (95% CI 0.02; 0.34) respectively. This indicated a change in nurses’ perceptions toward hospital performance indicators, feedback and training, thrombolysis evidence base, and its implementation along with emergency and ambulance service procedure, which was not present in physicians. Moreover, the sub-group analysis based on emergency and stroke care physicians was also no longer significant.

When comparing results between metropolitan and non-metropolitan hospitals, the domain 1 mean score in the non-metropolitan hospitals showed a significant mean increase of 0.26 (95% CI 0.17; 0.35; *p* < 0.001) indicating a change in staff perception related to hospital performance indicators, feedback, and training. In domain 2, the mean score in the metropolitan hospitals showed a significant mean increase of 0.25 (95% CI 0.04; 0.46; *p* < 0.05), indicating a change in staff perception related to the evidence supporting thrombolysis and its implementation. There were no significant differences in domains 3 and 4.

## Discussion

This is the first study to evaluate the effect of an implementation intervention aimed at increasing rates of intravenous thrombolysis on physicians’ and nurses’ perceptions utilizing a psychometrically tested tool for assessment on self-reported behavior. The assessment of the validity of the survey tool indicated that a four-factor structure with items loading on only one factor ≥ 0.4, minimal cross-loadings between items, and a structure that makes conceptual sense. Thus, the content validity of structure was supported. Moreover, all the domains had satisfactory internal consistency reliability, measured through Cronbach’s alpha; ranged 0.79–0.90. Therefore, the survey was considered an appropriate tool for assessing physicians’ and nurses’ perceptions. The TIPS intervention appeared to have some influence as would be expected according to the BCW framework, via strategies such as education and training. The intervention appeared to be effective in changing the perceptions of clinical staff in relation to their hospitals’ performance indicators, feedback and training, and their individual perceptions about the evidence base supporting post-stroke thrombolysis and its implementation. However, this appeared to be the case only for nurses.

Several studies have highlighted the use of constructive monitoring and feedback as a strategy for achieving positive changes in hospital-level performance [33, 34]. Moreover, identifying strategic goals is widely used as a means of enhancing organizational motivation, adherence, and autonomy, and in turn, improving processes of care [35, 36]. Hospital performance indicators such as those assessed in the survey can facilitate patient choice, can promote accountability, and finally can increase the quality of patient care [37]. In addition, stroke survivors are known to have complex needs and therefore, require the presence of a multidisciplinary team with specialized knowledge, skills, and experience in stroke [38]. The development and delivery of stroke-specific education are therefore of vital importance to the provision of high-quality stroke care and to improve outcomes for people who have experienced stroke. TIPS intervention, utilized a “monitoring-evaluation-feedback” strategy that involved site-based leaders where the primary change agents provided regular feedback and conducted monitoring to site champions at each hospital via phone calls throughout the intervention phase, and a knowledge translation strategy that provided web-based training modules, case monitoring, and problem-solving activities [17]. Such knowledge translation strategies have been demonstrated to improve health professionals’ perceptions of evidence-based approaches and their implementation [39, 40]. However, why this was more effective in nurses, as opposed to physicians, in our study is uncertain.

Failure of the TIPS intervention to change the perceptions of physicians may be due to limited statistical power (*n* = 74 physicians). However, the evidence does indicate that it can be challenging to change physicians’ perceptions about their clinical practice because they are long-standing and widely held [41, 42]. It is possible that physicians are more cautious in these potentially high-risk circumstances, given their level of responsibility for decision-making. Finally, an additional explanation may be that more nurses than physicians were engaged with the TIPS online learning modules made available to participants [17].

Hospital policies are often difficult to change as they involve complex systems [37, 38]. Given this complexity and a very limited focus on policy in the TIPS intervention, it is perhaps unsurprising that no change was found for the domain scores related to hospital policy and individual skills. While skills may be more amenable to change than policies, a ceiling effect may have been evident in relation to skills: According to results from the pre-intervention survey [26], most participants in the TIPS were skilled in post-stroke thrombolysis. For example, 98% could correctly assess stroke and 83% could correctly identify patients eligible for intravenous thrombolysis. The result from Grady et al. [25] was very similar, i.e., 98% of emergency physicians reported they could identify a stroke patient clearly, and 76% could identify patients eligible for intravenous thrombolysis. Finally, perceptions related to emergency services were not part of the TIPS intervention which accords with the null finding in relation to the domain score regarding perception toward emergency care.

Unfortunately, the study struggled with several limitations. As mentioned before, the number of participating physicians was low, which may have limited the study’s statistical power in relation to physician data, and the data were self-reported. The study was also not powered to evaluate the effect of the changes in perception at an individual site level. Therefore, the study suggests a need to explore in more depth (e.g., via more comprehensive mixed methods) the experience of the staff involved in implementation interventions and intervention uptake/adherence to developing better and sustainable intervention for the future.

## Conclusion

The TIPS intervention appeared to have more impact on changing the perception of nurses than physicians, particularly in the domains of hospitals’ performance, and feedback and training and perceptions about the thrombolysis evidence and its implementation. This further reinforces evidence about how challenging it is to change clinical practice and to effectively and efficiently bridge the evidence-practice gap.

## Supplementary information

**Additional file 1.** STROBE Statement.

**Additional file 2.** Supplement 1. Domains selected by factor analysis. Supplement 2. Domains selected by factor analysis. Supplement 3. Conceptual sense for the Factor structure.

## Data Availability

The datasets used and/or analyzed during the current study are available from the corresponding author on reasonable request.

## Abbreviations

AIS: Acute ischemic stroke
ASGC: Australian Standard Geographical Classification
NHMRC: National Health and Medical Research Council
NSW: New South Wales
TIPS: Thrombolysis ImPlementation in Stroke
