# Supplementary material for: Can a multicomponent multidisciplinary implementation package change physicians’ and nurses’ perceptions and practices regarding thrombolysis for acute ischemic stroke? An exploratory analysis of a cluster-randomized trial
Source: Implement Sci. 2019 Nov 27;14:98. doi: 10.1186/s13012-019-0940-0 (PMC6880372; doi:10.1186/s13012-019-0940-0)
Supplement: Supplementary file 2 — Additional file 2. Supplement 1. Domains selected by factor analysis. Supplement 2. Domains selected by factor analysis. Supplement 3. Conceptual sense for the Factor structure. [file 13012_2019_940_MOESM2_ESM.docx]

**Supplement 1:** Domains selected by factor analysis.

**Sample survey questionnaire**

Survey ID:

**Nurse Survey**

**Purpose of this survey:** We are interested in your views about the use of thrombolytic therapy with tissue plasminogen activator (tPA), or clot busters, for acute stroke care.

The information you give us by completing this survey will help us to identify potential barriers to the implementation of tPA in your hospital and will assist in developing service improvements for stroke care at your hospital.

Before you complete the survey, please read the information statement given to you and be sure you understand its contents. Completion and submission of this questionnaire will be considered ‘implied consent’ for your participation in this study.

The survey has 2 sections. It will take about 15 minutes to complete.

- **Section A** asks you to indicate your level of agreement regarding a number of statements concerning tPA use or stroke care in your hospital.
- **Section B** asks for some background information about you.

Please remember that any information you give us will remain confidential.

Should you have any questions or would like further information, please do not hesitate to contact the research team on 1800 084755.

***Thank you for taking the time to complete this survey***

**Section A: Your views on tPA** (*please circle)*

Please rate the extent to which you agree/disagree with the following statements by circling the appropriate number:

|  | Strongly Disagree | Disagree | Agree | Strongly Agree | *Not applicable* |
| --- | --- | --- | --- | --- | --- |
| **I can accurately identify:** |  |  |  |  |  |
| - Stroke patients | 1 | 2 | 3 | 4 | *5* |
| - Which stroke patients may be eligible for tPA | 1 | 2 | 3 | 4 | *5* |
|  |  |  |  |  |  |
| **This hospital has a policy for:** |  |  |  |  |  |
| - The management and care of stroke patients | 1 | 2 | 3 | 4 | *5* |
| - Rapid referral of suspected stroke patients from ED to stroke specialists | 1 | 2 | 3 | 4 | *5* |
| - Rapid access to imaging for suspected stroke patients | 1 | 2 | 3 | 4 | *5* |
| - Administration of tPA when appropriate | 1 | 2 | 3 | 4 | *5* |
|  |  |  |  |  |  |
| **This hospital has:** |  |  |  |  |  |
| - Indicators for quality of stroke care | 1 | 2 | 3 | 4 | *5* |
| - Goals for improving performance on stroke care indicators | 1 | 2 | 3 | 4 | *5* |
|  |  |  |  |  |  |
| **Increasing appropriate use of tPA will:** |  |  |  |  |  |
| - Save lives | 1 | 2 | 3 | 4 | *5* |
| - Improve the odds of independent survival for stroke patients | 1 | 2 | 3 | 4 | *5* |
| - Increase the number of patients with complications or poor outcomes | 1 | 2 | 3 | 4 | *5* |
|  |  |  |  |  |  |
| **The evidence underpinning tPA use:** |  |  |  |  |  |
| - Is strong when administered within 3 hours of stroke onset | 1 | 2 | 3 | 4 | *5* |
| - Is strong when administered within 4.5 hours of stroke onset | 1 | 2 | 3 | 4 | *5* |
| - Indicates that the benefits outweigh the risks, if the treatment protocol is followed | 1 | 2 | 3 | 4 | *5* |
| - Has methodological flaws | 1 | 2 | 3 | 4 | *5* |
| - Is based on high quality meta-analyses | 1 | 2 | 3 | 4 | *5* |
| - Indicates a theoretical benefit that is difficult to achieve in practice | 1 | 2 | 3 | 4 | *5* |
|  | Strongly Disagree | Disagree | Agree | Strongly Agree | *Not applicable* |
| **The implementation of tPA in practice is limited because:** |  |  |  |  |  |
| - Of difficulties in using the SITS registry | 1 | 2 | 3 | 4 | *5* |
| - Intracranial hemorrhage rates will be higher in practice than in the trials | 1 | 2 | 3 | 4 | *5* |
|  |  |  |  |  |  |
| **Respected and influential members of this hospital:** |  |  |  |  |  |
| - Advocate for the use of tPA | 1 | 2 | 3 | 4 | *5* |
| - Actively demonstrate best practice stroke care to all staff | 1 | 2 | 3 | 4 | *5* |
|  |  |  |  |  |  |
| **Respected and influential members of this hospital monitor:** |  |  |  |  |  |
| - My performance of stroke care | 1 | 2 | 3 | 4 | *5* |
| - The hospitals’ performance on key stroke care indicators | 1 | 2 | 3 | 4 | *5* |
| - Actions which are inconsistent with guideline care for stroke patients | 1 | 2 | 3 | 4 | *5* |
| - The proportion of eligible stroke patients who receive tPA | 1 | 2 | 3 | 4 | *5* |
|  |  |  |  |  |  |
| **This hospital has a system in place for:** |  |  |  |  |  |
| - Reviewing outcomes of quality improvement plans for stroke care | 1 | 2 | 3 | 4 | *5* |
| - Implementing action plans for improving performance for stroke care | 1 | 2 | 3 | 4 | *5* |
| - Entering data from all patients treated with tPA into a central register | 1 | 2 | 3 | 4 | *5* |
|  |  |  |  |  |  |
| **Emergency/ ambulance services:** |  |  |  |  |  |
| - Are able to quickly and correctly identify potential stroke patients | 1 | 2 | 3 | 4 | *5* |
| - Use a recognised tool (eg FAST) for identifying potential stroke patients | 1 | 2 | 3 | 4 | *5* |
| - Have procedures for providing early notification of a stroke patient to the Emergency Department/ Stroke Care Unit | 1 | 2 | 3 | 4 | *5* |
| - Routinely divert potential stroke patients to hospitals which provide thrombolysis | 1 | 2 | 3 | 4 | *5* |
|  |  |  |  |  |  |
|  | Strongly Disagree | Disagree | Agree | Strongly Agree | *Not applicable* |
| **I am regularly given:** |  |  |  |  |  |
| - Individual performance feedback following relevant cases of acute stroke | 1 | 2 | 3 | 4 | *5* |
|  |  |  |  |  |  |
| **This hospital regularly receives feedback on:** |  |  |  |  |  |
| - Its performance on stroke care indicators | 1 | 2 | 3 | 4 | *5* |
|  |  |  |  |  |  |
| **If I do not follow stroke care and tPA protocol, there are negative consequences for:** |  |  |  |  |  |
| - The patient | 1 | 2 | 3 | 4 | *5* |
| - The hospital | 1 | 2 | 3 | 4 | *5* |
| - Me | 1 | 2 | 3 | 4 | *5* |
|  |  |  |  |  |  |
| **I have:** |  |  |  |  |  |
| - Seen tPA administered on multiple occasions | 1 | 2 | 3 | 4 | *5* |
| - Received interactive training in best practice care of stroke patients | 1 | 2 | 3 | 4 | *5* |
| - Undergone competency-based assessment for stroke protocol(s) | 1 | 2 | 3 | 4 | *5* |
| - Individual performance goals related to stroke care | 1 | 2 | 3 | 4 | *5* |
|  |  |  |  |  |  |
| **This hospital has:** |  |  |  |  |  |
| - The capacity and a system to make a bed available quickly for a stroke patient | 1 | 2 | 3 | 4 | *5* |
|  |  |  |  |  |  |
| **I regularly:** |  |  |  |  |  |
| - Have the opportunity to develop my skills in stroke care | 1 | 2 | 3 | 4 | *5* |
| - Care for acute stroke patients | 1 | 2 | 3 | 4 | *5* |
| - Have the opportunity to care for stroke cases of varying complexity | 1 | 2 | 3 | 4 | *5* |
|  |  |  |  |  |  |
| **To help me follow stroke care protocol there are:** |  |  |  |  |  |
| - Checklists/ decision aids to help me identify and triage a possible stroke case | 1 | 2 | 3 | 4 | *5* |
| - Checklists/ decision aids to help me identify stroke patients eligible for tPA | 1 | 2 | 3 | 4 | *5* |
|  |  |  |  |  |  |
|  |  |  |  |  |  |
|  |  |  |  |  |  |
|  | Strongly Disagree | Disagree | Agree | Strongly Agree | *Not applicable* |
|  |  |  |  |  |  |
| **At all times I have immediate access to:** |  |  |  |  |  |
| - Advice by from a senior colleague in providing care for stroke | 1 | 2 | 3 | 4 | *5* |
| - Brain imaging facilities and staff trained to interpret images | 1 | 2 | 3 | 4 | *5* |

**Please list any additional barriers you are encountering within your department in the administration of tPA to all eligible ischaemic stroke patients**

**______________________________________________________________________________________________________________________________________________________**

**Please list any additional barriers you are encountering outside your department (e.g. other hospital departments, emergency services, etc.) in achieving the administration of tPA to all eligible ischaemic stroke patients**

**Section B: About You and Your Workplace** (please tick)

**1. I am**

□ Male

□ Female

**2. How old are you?**

**________years**

**3. How many years have you worked in emergency care/ stroke care?**

□ 5 years or less

□ 5-10 years

□ 11-15 years

□ 16 years or more

**4. What is your role within the hospital?**

□ Registered Nurse

□ Enrolled Nurse

□ Emergency Nurse Specialist

□ Stroke Nurse Specialist

□ Other, please specify ___________________________________

**5. Does your role at the hospital include responsibility for entering stroke cases into databases such as TIPS or the Stroke Foundation Audit?**

□ Yes

□ No

**6. Would any of the following influence your views on the use of tPA in acute stroke?**

Please select as many as apply.

□ Guidance from a professional colleague

□ Guidance from a visiting expert Professor

□ Additional clinical trials of tPA

□ Research conducted by your department

□ None of the above

□ Other, please specify _________________________

**7. Does the hospital have arrangements in place for pre-arrival notification of stroke patients from the ambulance service?**

□ Yes – Notification goes to the emergency department/ emergency specialists

□ Yes – Notification goes to the Stroke Care Unit (SCU)/ stroke specialists

□ No

□ Other

**8. Please estimate on average how many ischaemic stroke patients are seen by the emergency department during a fortnight of your shifts.**

_________patients

**9. Of the stroke patients seen by the emergency department during a fortnight of your shifts, please estimate the proportion that are referred to a stroke care unit or neurology department.**

________%

**10. Please estimate approximately what proportion of eligible ischaemic stroke patients are currently treated with thrombolysis at the hospital.**

________%

**Supplement 2:** Domains selected by factor analysis.

| **Domain 1** | **Domain** |
| --- | --- |
| Hospital has goals for improving performance on stroke care indicators | Domain 1: Hospital performance indicators, feedback and training |
| Respected and influential members of hospital actively demonstrate best practice stroke care to all staff |  |
| Respected and influential members of hospital monitor performance of stroke care |  |
| Respected and influential members of hospital monitor the hospitals’ performance on key stroke care indicators |  |
| Respected and influential members of hospital monitor actions which are inconsistent with guideline care for stroke patients |  |
| Respected and influential members of hospital monitor the proportion of eligible stroke patients who receive tPA |  |
| Hospital has a policy for reviewing outcomes of quality improvement plans for stroke care |  |
| Hospital has a policy for implementing action plans for improving performance for stroke care |  |
| Hospital has a policy for entering data from all patients treated with tPA into a central register |  |
| Hospital regularly given individual performance feedback following relevant cases of acute stroke |  |
| Hospital regularly receives feedback on its performance on stroke care indicators |  |
| Have undergone competency based assessment on stroke protocols |  |
| Have individual performance goals related to stroke care |  |
| Have the opportunity to care for stroke cases of varying complexity |  |
| **Domain 2** |  |
| Increasing appropriate use of tPA will save lives | Domain 2: Personal perceptions about the thrombolysis evidence base and its implementation |
| Increasing appropriate use of tPA will improve the odds of independent survival for stroke patients |  |
| The evidence underpinning tPA use is strong when administered within 3 hours of stroke onset |  |
| The evidence underpinning tPA use is strong when administered within 4.5 hours of stroke onset |  |
| The evidence underpinning tPA use indicates that the benefits outweigh the risks, if the treatment protocol is followed |  |
| The evidence underpinning tPA use has methodological flaws |  |
| The evidence underpinning tPA use is based on high quality meta-analyses |  |
| If I do not follow stroke care and tPA protocol, there are negative consequences for the patient |  |
| If I do not follow stroke care and tPA protocol, there are negative consequences for: The hospital |  |
| **Domain 3** | Domain 3: Personal stroke skills and hospital stroke care policies |
| I can identify stroke patient accurately |  |
| I can identify patients eligible for IV-tPA |  |
| Hospital has policy for management and care for stroke patient |  |
| Hospital has policy for referral patients from ED to stroke specialist |  |
| Hospital has policy for rapid access to imaging for suspected cases |  |
| Hospital has policy for IV-tPA administration when needed |  |
| **Domain 4** |  |
| Emergency/ ambulance services are able to quickly and correctly identify potential stroke patients | Domain 4: Emergency and ambulance procedure |
| Emergency/ ambulance services use a recognised tool (e.g. FAST) for identifying potential stroke patients |  |
| Emergency/ ambulance services have procedures for providing early notification of a stroke patient to the Emergency Department/ Stroke Care Unit |  |
| Emergency/ ambulance services routinely divert potential stroke patients to hospitals which provide thrombolysis |  |

**Supplement 3:** Conceptual sense for the Factor structure

1. The items under the domain “Hospital performance indicators, feedback, and training” highlights the activity and policy set by the hospital leaders and administrators to supervise and monitor the process of stroke care.
2. The items under the domain “Personal perceptions about the thrombolysis evidence base and its implementation” identifies the knowledge level of staff on thrombolysis and its implementation.
3. The items under the domain “Personal stroke skills and hospital stroke care policies” checks the level of the staff skills and hospital policies required to reduce the in-hospital process time for thrombolysis.
4. The items under the domain “Emergency and ambulance procedure” measures the performance of the pre-hospital emergency system of stroke care.
